# Supplementary material for: Synthesis, Transfer, and Gas Separation Characteristics of MOF-Templated Polymer Membranes
Source: Membranes (Basel). 2019 Sep 20;9(10):124. doi: 10.3390/membranes9100124 (PMC6835741; doi:10.3390/membranes9100124)
Supplement: Supplementary file 1 [file membranes-09-00124-s001.pdf]

Synthesis, Transfer, and Gas Separation Characteristics of MOF-Templated Polymer Membranes

Sophia Schmitt [1], Sergey Shishatskiy [2], Peter Krolla [1], Qi An [1], Salma Begum [1], Alexander Welle [1,3], Tawheed Hashem [1,8], Sylvain Grosjean [4,5], Volker Abetz [2,6], Stefan Bräse [4,7], Christof Wöll [1] and Manuel Tsotsalas [1,4,*]

1. Institute of Functional Interfaces (IFG), Karlsruhe Institute of Technology (KIT), Hermann-von Helmholtz-Platz 1, 76344 Eggenstein-Leopoldshafen, Germany; Sophia.Schmitt@web.de (So.Sc.); peter.krolla@kit.edu (P.K.); qi.an@kit.edu (Q.A.); salma.begum@kit.edu (Sa.Be.); alexander.welle@kit.edu (A.W.); tawheed.hashem@kit.edu (T.H.); christof.woell@kit.edu (C.W.)
2. Institute of Polymer Research, Helmholtz-Zentrum Geesthacht (HZG), Max-Planck-Street 1, 21502 Geesthacht, Germany; sergey.shishatskiy@hzg.de (Se.Sh.); volker.abetz@hzg.de (V.A.)
3. Karlsruhe Nano Micro Facility (KNMF), Karlsruhe Institute of Technology (KIT), Hermann-von-Helmholtz-Platz 1, 76344 Eggenstein-Leopoldshafen, Germany
4. Institute for Organic Chemistry (IOC), Karlsruhe Institute of Technology (KIT), Fritz-Haber-Weg 6, 76131 Karlsruhe, Germany; sylvain.grosjean@kit.edu (S.G.); stefan.braese@kit.edu (St.Br.)
5. Soft Matter Synthesis Lab, Institute of Biological Interfaces 3 (IBG3), Karlsruhe Institute of Technology (KIT), Hermann-von Helmholtz-Platz 1, 76344 Eggenstein-Leopoldshafen, Germany
6. Institute of Physical Chemistry, University of Hamburg, Martin-Luther-King-Platz 6, 20146 Hamburg, Germany
7. institute of Toxicology and Genetics (ITG), Karlsruhe Institute of Technology (KIT), Hermann-von Helmholtz-Platz 1, 76344 Eggenstein-Leopoldshafen, Germany
8. Institute of Physics and Technology, International X-ray Optics Lab, National Research Tomsk Polytechnic University (TPU), 30 Lenin ave., Tomsk 634050, Russia

* Correspondence: manuel.tsotsalas@kit.edu

Received: 26 August 2019; Accepted: 17 September 2019; Published: date

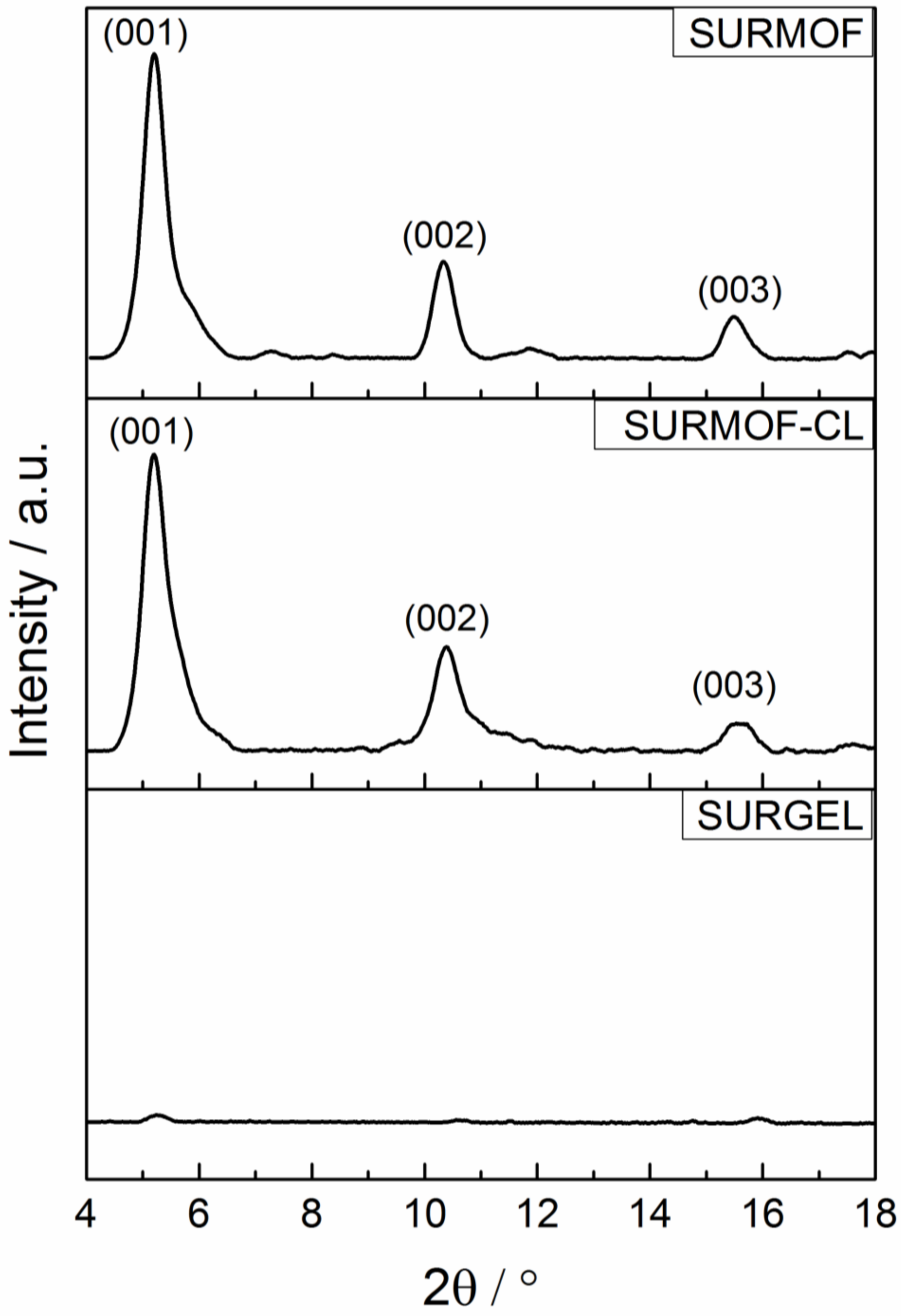

Figure S1: Corresponding X-ray diffraction (XRD) data of SURMOF samples before the cross-linking reaction (top), after the cross-linking (middle), and after metal removal and conversion to SURGEL. Please note that the data was recorded on a reference sample to avoid overlay with the diffraction peaks of the mica substrate.

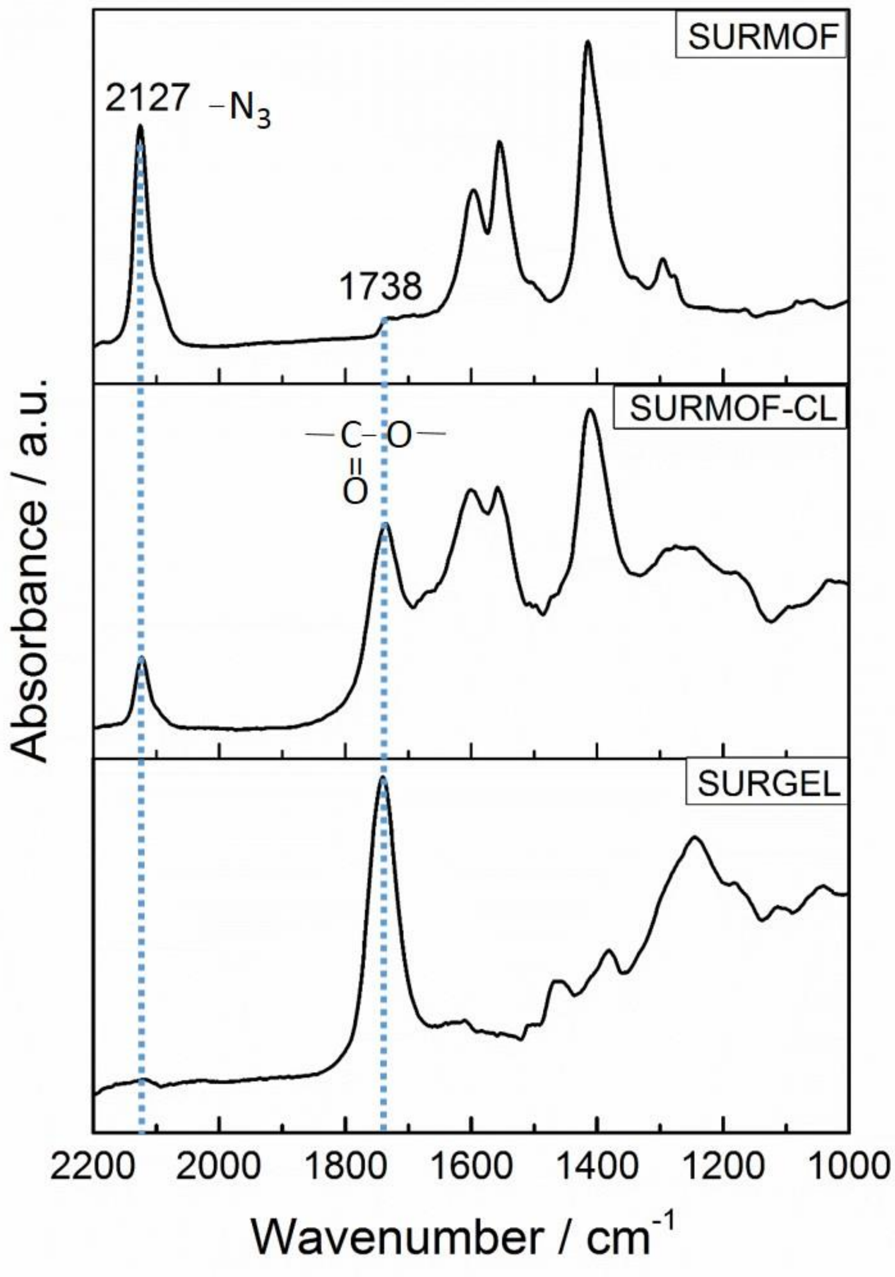

Figure S2: Corresponding IRRAS spectra of SURMOF before the cross-linking reaction (top), after the cross-linking (middle), and after metal removal and conversion to SURGEL.

Gas permeance data for the PDMS/PAN reference membrane.

Temperature [°C]	1/T [1/K]		He	H2	CO2	Ar	O2	N2	CH4	C2H6
30	3.30E-03		1.19	1.89	4.76	1.02	1.07	0.458	1.23	2.22
40	3.19E-03		1.45	2.28	4.64	1.19	1.24	0.555	1.42	2.45
50	3.09E-03		1.73	2.66	4.98	1.36	1.42	0.657	1.64	2.61
60	3.00E-03		2.07	3.15	5.23	1.57	1.62	0.783	1.88	2.86
70	2.91E-03		2.42	3.62	5.49	1.79	1.83	0.919	2.14	3.09

Gas permeance data for the SURGEL/PDMS/PAN membrane.

Temperature [°C]	1/T [1/K]	Area not covered with MOF	He	H2	CO2	Ar	O2	N2	CH4	C2H6
30	3.30E-03	0.023	0.306	0.177	0.0161	0.0019	0.0039	0.0021	0.0015	0.0013
40	3.19E-03	0.023	0.387	0.243	0.0369	0.0051	0.0064	0.0027	0.0051	0.0014
50	3.09E-03	0.023	0.487	0.297	0.0507	0.0070	0.0105	0.0036	0.0079	0.0093
60	3.00E-03	0.023	0.658	0.414	0.0695	0.0172	0.0171	0.0269	0.0272	0.0107
70	2.91E-03	0.023	0.888	0.525	0.0962	0.0394	0.0425	0.0455	0.0704	0.0353

Gas permeance data for the por-PMMA/SURGEL/PDMS/PAN membrane

Temperature [°C]	1/T [1/K]	Area not covered with MOF	He	H2	CO2	Ar	O2	N2	CH4	C2H6
30	3.30E-03	0.019	0.105	0.0588	0.0012	0.0017	0.0024	0.0021	0.0016	0.0003
40	3.19E-03	0.019	0.125	0.0712	0.0053	0.0023	0.0027	0.0030	0.0023	0.0007
50	3.09E-03	0.019	0.161	0.0904	0.0076	0.0052	0.0052	0.0058	0.0052	0.0033
60	3.00E-03	0.019	0.216	0.124	0.0154	0.0121	0.0134	0.0164	0.0144	0.0096
70	2.91E-03	0.019	0.264	0.159	0.0264	0.0199	0.0220	0.0537	0.0207	0.0189

Table S1: Measured gas permeances for each gas at the temperatures 30°C, 40°C, 50°C, 60°C, and 70°C. Top: reference membrane (PDMS/PAN); Middle: SURGEL/PDMS/PAN TFC membrane; Bottom: por-PMMA/SURGEL/PDMS/PAN TFC membrane.
